# Integrative analysis of clinicopathological features defines novel prognostic models for mantle cell lymphoma in the immunochemotherapy era: a report from The North American Mantle Cell Lymphoma Consortium

**DOI:** 10.1186/s13045-023-01520-7

**Published:** 2023-12-16

**Authors:** Julie M. Vose, Kai Fu, Lu Wang, Adnan Mansoor, Douglas Stewart, Hongxia Cheng, Lynette Smith, Ji Yuan, Hina Naushad Qureishi, Brian K. Link, Melissa H. Cessna, Paul M. Barr, Brad S. Kahl, Matthew S. Mckinney, Nadia Khan, Ranjana H. Advani, Peter Martin, Andre H. Goy, Tycel J. Phillips, Amitkumar Mehta, Manali Kamdar, Michael Crump, Barbara Pro, Christopher R. Flowers, Caron A. Jacobson, Sonali M. Smith, Deborah M. Stephens, Veronika Bachanova, Zhaohui Jin, Shishou Wu, Francisco Hernandez-Ilizaliturri, Pallawi Torka, Andrea Anampa-Guzmán, Farshid Kashef, Xing Li, Sunandini Sharma, Timothy C. Greiner, James O. Armitage, Matthew Lunning, Dennis D. Weisenburger, Robert G. Bociek, Javeed Iqbal, Guohua Yu, Chengfeng Bi, the North American Mantle Cell Lymphoma Consortium

**Affiliations:** 1 Division of Oncology and Hematology, Department of Internal Medicine, University of Nebraska Medical Center, Nebraska Medicine Fred and Pamela Buffett Cancer Center, 505 S 45Th St, Omaha, NE 68105 USA; 2 Department of Pathology, Roswell Park Comprehensive Cancer Center, Buffalo, NY USA; 3 Department of Pathology and Microbiology, University of Nebraska Medical Center, Omaha, NE USA; 4 School of Pharmacy, Key Laboratory of Molecular Pharmacology and Drug Evaluation (Yantai University), Ministry of Education, Yantai University, Yantai, China; 5 Department of Pathology and Laboratory Medicine, University of Calgary, Calgary, Canada; 6 Departments of Oncology and Medicine, University of Calgary, Calgary, Canada; 7 Department of Pathology, Shandong Provincial Hospital Affiliated to Shandong First Medical University, Jinan, Shandong Province China; 8 Department of Biostatistics, University of Nebraska Medical Center, Omaha, NE USA; 9 Department of Laboratory Medicine and Pathology, Mayo Clinic, Rochester, MN USA; 10 Department of Internal Medicine, University of Iowa Hospitals & Clinics, Iowa City, Iowa, USA; 11 Department of Pathology, Intermountain Medical Center, Murray, UT USA; 12 Department of Medicine, University of Rochester Medical Center, Rochester, NY USA; 13 Department of Medicine, Oncology Division, Washington University School of Medicine in St. Louis, St. Louis, MO USA; 14 Division of Hematologic Malignancies and Cellular Therapy, Department of Medicine, Duke University Medical Center, Durham, NC USA; 15 Department of Hematology/Oncology, Department of Hematology/Oncology, Fox Chase Cancer Center, Philadelphia, PA USA; 16 Division of Oncology, Stanford Cancer Institute, Stanford, CA USA; 17 Division of Hematology and Oncology, Weill Cornell Medical College, New York, NY USA; 18 John Theurer Cancer Center at Hackensack University Medical Center, Hackensack, NJ USA; 19 Department of Internal Medicine, Division of Hematology/Oncology, University of Michigan Medical School, Ann Arbor, MI USA; 20 O’Neal Comprehensive Cancer Center, University of Alabama at Birmingham, Birmingham, AL USA; 21 Division of Hematology, University of Colorado, Denver, CO USA; 22 Division of Medical Oncology and Hematology, Princess Margaret Cancer Centre - University Health Network, Toronto, ON Canada; 23 Division of Hematology and Oncology, Department of Medicine, Northwestern University Feinberg School of Medicine, Chicago, USA; 24 Division of Cancer Medicine, Department of Lymphoma-Myeloma, MD Anderson Cancer Center, Houston, TX USA; 25 Department of Medical Oncology, Dana-Farber Cancer Institute, Harvard Medical School, Boston, MA USA; 26 Section of Hematology/Oncology, Department of Medicine, University of Chicago, Chicago, IL USA; 27 Huntsman Cancer Institute at University of Utah, Salt Lake City, UT USA; 28 Division of Hematology, Oncology and Transplantation, Department of Medicine, University of Minnesota, Minneapolis, MN USA; 29 Department of Oncology, Mayo Clinic, Rochester, MN USA; 30 Department of Pathology, Affiliated Yantai Yuhuangding Hospital, Qingdao University, No.20 Yuhuangding East Road, Yantai, 264000 China; 31 Department of Medicine, Roswell Park Comprehensive Cancer Center, Buffalo, NY USA; 32 Department of Medicine, Memorial Sloan Kettering Cancer Center, New York, NY USA; 33 Department of Pathology, University at Buffalo, Buffalo, NY USA

## Abstract

**Background:**

Patients with mantle cell lymphoma (MCL) exhibit a wide variation in clinical presentation and outcome. However, the commonly used prognostic models are outdated and inadequate to address the needs of the current multidisciplinary management of this disease. This study aims to investigate the clinical and pathological features of MCL in the immunochemotherapy era and improve the prognostic models for a more accurate prediction of patient outcomes.

**Methods:**

The North American Mantle Cell Lymphoma Project is a multi-institutional collaboration of 23 institutions across North America to evaluate and refine prognosticators for front-line therapy. A total of 586 MCL cases diagnosed between 2000 and 2012 are included in this study. A comprehensive retrospective analysis was performed on the clinicopathological features, treatment approaches, and outcomes of these cases. The establishment of novel prognostic models was based on in-depth examination of baseline parameters, and subsequent validation in an independent cohort of MCL cases.

**Results:**

In front-line strategies, the use of hematopoietic stem cell transplantation was the most significant parameter affecting outcomes, for both overall survival (OS, *p* < 0.0001) and progression-free survival (PFS, *p* < 0.0001). P53 positive expression was the most significant pathological parameter correlating with inferior outcomes (*p* < 0.0001 for OS and *p* = 0.0021 for PFS). Based on the baseline risk factor profile, we developed a set of prognostic models incorporating clinical, laboratory, and pathological parameters that are specifically tailored for various applications. These models, when tested in the validation cohort, exhibited strong predictive power for survival and showed a stratification resembling the training cohort.

**Conclusions:**

The outcome of patients with MCL has markedly improved over the past two decades, and further enhancement is anticipated with the evolution of clinical management. The innovative prognostic models developed in this study would serve as a valuable tool to guide the selection of more suitable treatment strategies for patients with MCL.

**Supplementary Information:**

The online version contains supplementary material available at 10.1186/s13045-023-01520-7.

## Introduction

Mantle cell lymphoma (MCL), accounting for 3–10% of non-Hodgkin lymphoma, is a mature B cell lymphoma characterized by cyclin D1 rearrangement. MCL occurs mostly in the lymph nodes with frequent extranodal involvement, and most patients have advanced-stage disease at the time of the first diagnosis [1, 2]. MCL has unique biological characteristics, and although often sensitive to therapy at first, relapses are frequent and most patients eventually experience disease progression following conventional treatment. Over the last two decades, along with the broad application of immunochemotherapy, hematopoietic stem cell transplantation (HSCT), and small molecule inhibitors, the clinical outcome of MCL patients has improved significantly [3]. Due to the wide variability in clinical presentation and outcome, several risk stratification methods have been developed for predicting the prognosis of MCL patients. At present, the most commonly used model is the Mantle Cell Lymphoma International Prognostic Index (MIPI) [4], including the upgraded MIPI-c [5]. However, several disadvantages have been encountered in the practical application of this stratification method, which led to inconsistent results in validation studies [6]. First, MIPI was developed in the chemotherapy era, and although subsequent studies validated its efficacy in patients undergoing immunochemotherapy [7, 8], a refined stratification system using recent cohorts may exhibit superior predictive power. Second, MIPI was originally developed solely on clinical and laboratory parameters, without considering pathological features. While the MIPI-c algorithm has incorporated the Ki-67 index, it is crucial to also consider the potential impact of other pathological features. This is particularly important for p53 overexpression, as emerging evidence has linked its presence with poorer outcomes. Therefore, new prognostic models derived from an integrative analysis of major clinical, laboratory, and pathological parameters may offer a more comprehensive and accurate risk assessment. Third, MIPI was established based on overall survival (OS), whereas more accurate prediction of disease relapse upon front-line therapy is in demand given that new therapies, such as bruton tyrosine kinase (BTK) inhibitors and chimeric antigen receptor T cell (CAR-T) therapy tend to play more important roles in the MCL treatment. Therefore, a prognostic model established on progression-free survival (PFS) is of practical value in assisting the selection of treatment strategies.

The North American Mantle Cell Lymphoma Project (NAMCLP) was organized with the goal of enhancing the clinical management of MCL patients. In this study, we analyze the clinicopathological data of a large cohort of MCL patients and establish a new set of prognostic models with high prognostic power, functioning not only from the general perspective but also in an age-defined manner (< 65 years and ≥ 65 years, respectively).

## Patients and methods

### Patients

An initial analytic cohort of 586 MCL cases diagnosed between January 2000 and December 2012 was recruited from 23 participating institutions across North America. An independent validation cohort of 185 cases diagnosed between January 2000 and December 2017 was subsequently recruited from University of Nebraska Medical Center (UNMC), Roswell Park Comprehensive Cancer Center, and University of Calgary. To be included, cases had to have documented information on clinical examination and management. The study protocol was approved by the local ethics committees of participating institutions. The clinical information was collected for all cases, and pathology review was conducted in cases with available material.

### Pathologic assessment

For the analytic cohort, the pathologic features including growth pattern, cytologic features, and immunoglobulin light chain restriction (ILCR), as well as the immunohistochemical (IHC) staining for CD5, CD23, Ki-67, and p53, were reviewed by three experienced hematopathologists. The diffuse growth pattern was defined as 100% diffuse growth, and tumor cytology referred to classical or blastoid/pleomorphic subtypes. Needle biopsy samples were not included in the morphology subclassification due to their limited tissue representation. All p53 staining (antibody clone: DO-7, Ventana, USA) was performed at the UNMC. The cutoff value for Ki-67 followed the previously established criteria of 30% [6, 9], whereas p53 staining was assessed by a semi-quantitative scoring method as described previously [10]. Briefly, the intensity of nuclear p53 staining (none 0, weak 1, medium 2, and strong 3) was multiplied by the percentage of positive tumor cells. Summing up each faction led to the final score with the positive cutoff setting as ≥ 0.9.

### Statistical methods

To validate the MIPI and MIPI-c stratification, Kaplan–Meier OS (calculated from the date of diagnosis to death from any cause) and PFS (calculated from the date of diagnosis to relapse or death from any cause) curves for stratified risk groups were calculated and compared by the log-rank test. The prognostic relevance of the candidate prognostic factors was evaluated using univariate Cox regression for OS and PFS. Subsequently, multiple Cox regression with backward variable selection was performed to identify the most optimized combination of relevant prognostic factors, as well as the final Cox regression model. The proportional hazards assumption for the final model was checked using scaled Schoenfeld residuals [11, 12]. Factors in violation of that assumption or with substantial multivariate missing values were excluded from the multiple regression. Prognostic groups were defined by categorizing the prognostic scores derived from the final Cox regression model. Two optimal cutpoints for the overall evaluation and one for age-defined assessments maximizing the log-rank statistic were identified following the “minimal *P* value approach” [13]. *P* values for the log-rank statistic were adjusted for multiple testing by the Bonferroni method. Internal validation for the log-rank test statistic and Harrell’s C-index was performed by applying the refined bootstrap described by Efron [14]. Statistical analyses were performed using R version 4.1.1.

## Results

### Clinical, laboratory, and pathological characteristics

The male-to-female ratio of this cohort of patients was 3.6:1, and 89% of the patients were Caucasian. The median age was 64 years (range 24–104 years, Additional file 1: Fig. S1), and thus, using age 65 as the cutoff, we divided the patients into younger (< 65) and older cohorts (≥ 65). At diagnosis, 93% of the patients had an ECOG performance score < 2, 24% presented with B symptoms, 73% had extranodal involvement, and 89% had stage III-IV disease. Elevated WBC was present in 22%, elevated serum lactate dehydrogenase (LDH) in 40%, anemia in 35%, thrombocytopenia in 15%, and circulating tumor cells (CTCs, determined by flow cytometry) in 39% of the patients. A central pathology review was conducted on a total of 315 cases. Among these, the diffuse growth pattern was observed in 41%, and the blastoid/polymorphic variant was present in 17% of the cases. CD5 and CD23 were positive in 90% and 13% of the cases, respectively; 28% had a p53 score ≥ 0.9 and 33% had a Ki-67 value ≥ 30%. All characteristics are listed in Table 1.

**Table 1:** Baseline and Treatment Information for The Analytical Cohort of MCL Cases (*n* = 586)

Parameter	Quantity	n	%	Parameter	Quantity	*n*	%
Age ≥ 65y	354	578	61.2	Lymphocytes (> 5˟10^3^/μL)	93	457	20.4
Male	455	581	78.3	Anemia	176	508	34.7
Caucasian	452	507	89.2	Platelets (≤ 100 k/μL)	78	510	15.3
ECOG Performance < 2	502	538	93.3	Circulating tumor cells	134	345	38.8
B symptoms presented	131	538	24.3	Largest tumor diameter (≥ 3 cm)	230	444	51.8
Nodal involvement only	151	550	27.5	Blastoid or Pleomorphic	38	222	17.1
Extranodal involvement				Diffuse growth pattern	91	223	40.8
*Tonsil*	*37*	*395*	*9.4*	CD5 positive	252	279	90.3
*Colon*	*72*	*490*	*14.7*	CD23 positive	25	186	13.4
*Liver*	*22*	*490*	*4.5*	Kappa light chain restriction	59	139	42.4
*Small intestine*	*50*	*490*	*10.2*	p53 (≥ 0.9 points)	77	273	28.2
*Spleen*	*134*	*490*	*27.3*	Ki-67 (≥ 30%)	100	306	32.7
*Stomach*	*24*	*490*	*4.9*	SOX11 (≥ 10%)	181	202	89.6
*Digestive tract*	*98*	*490*	*20.0*	Chemotherapy regimens			
Limited to one site	29	527	5.5	*Cytarabine-based*	*159*	*503*	*31.6*
AAS III-IV	488	548	89.1	*Anthracycline-based*	*244*	*503*	*48.5*
Elevated LDH	179	453	39.5	*Purine analogue-based*	*69*	*503*	*13.7*
Elevated Beta-MG	93	159	58.5	*Others*	*31*	*503*	*6.2*
Clonal Ig	23	105	21.9	Rituximab maintenance	96	485	19.8
WBCs (> 11˟10^3^/μL)	116	523	22.0	HSCT	167	511	32.7
Neutrophils (> 10˟10^3^/μL)	7	455	1.5	Auto-SCT vs Allo-SCT	133/33	166	80.1/19.9

*AAS* Ann Arbor stage, *Beta-MG* beta-microglobulin; *LDH* lactate dehydrogenase; *SCT* stem cell transplantation; and *WBC* white blood cell

### Treatment and outcome

**Fig. 1:**
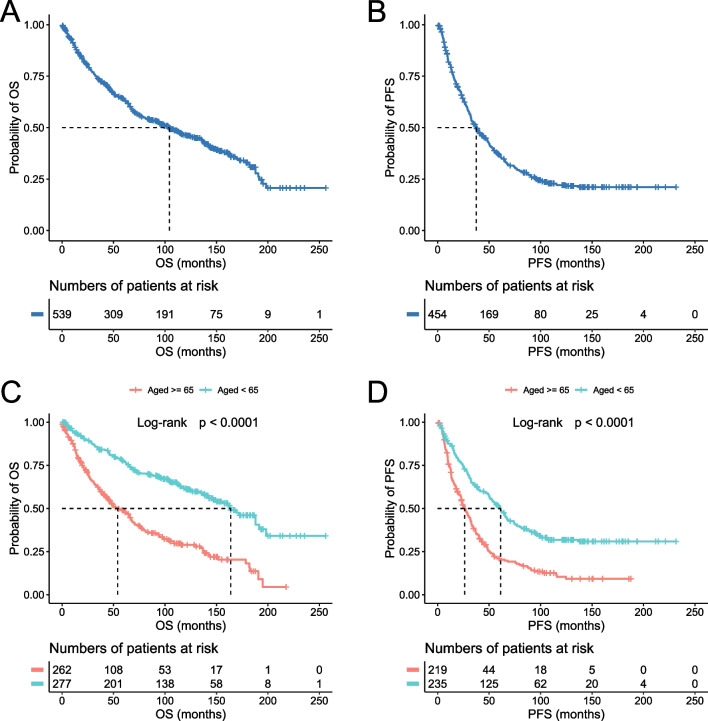
Survival rates of the analytic cohort. The OS and PFS of the entire cohort (**A** and **B**) and the age-defined cohorts (**C** and **D**) are shown

**Fig. 2:**
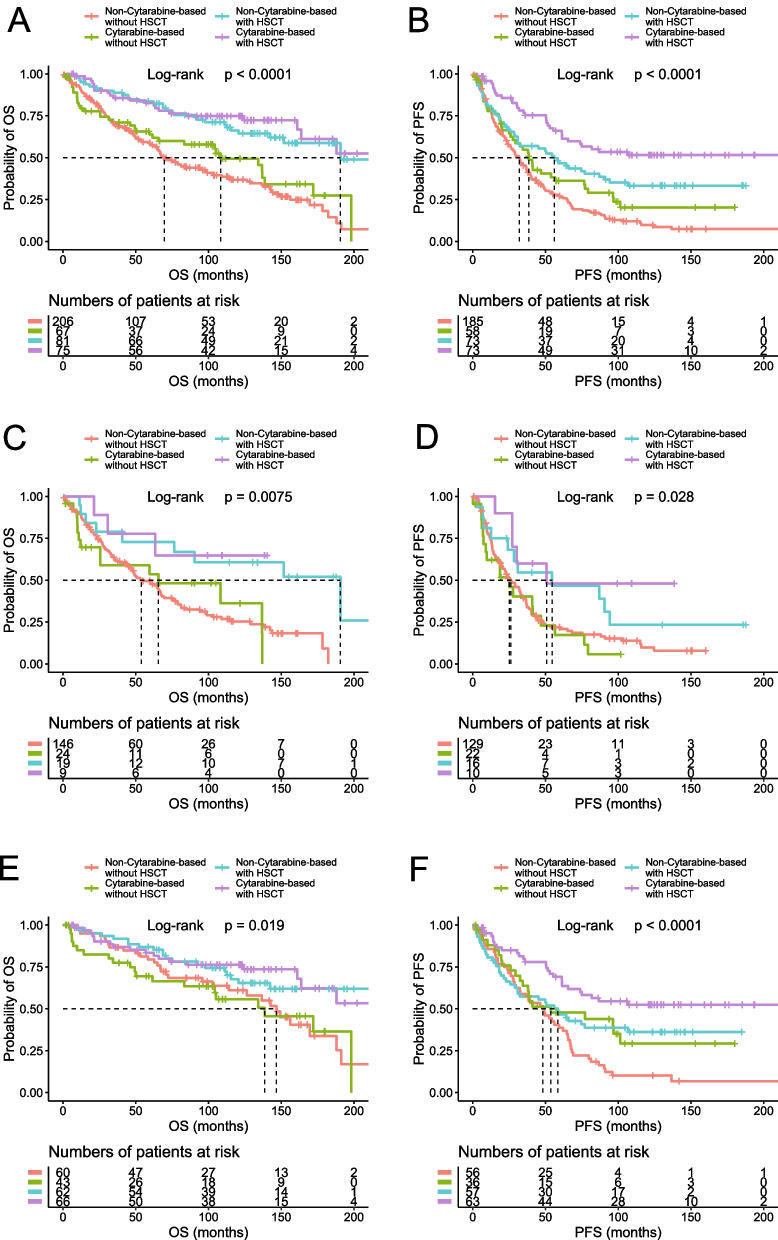
Treatment and survival outcomes. The patients were divided into four groups: cytarabine-based chemotherapy with or without HSCT, and non-cytarabine-based chemotherapy with or without HSCT. The OS and PFS of the entire cohort (**A** and **B**), the older cohort (**C** and **D**), and the younger cohort (**E** and **F**) are shown

**Fig. 3:**
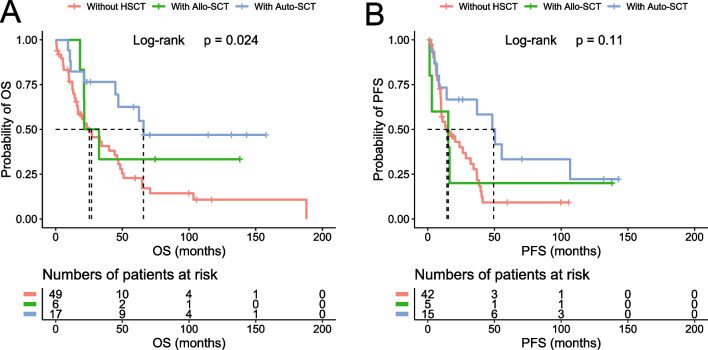
Impact of HSCT on the survival of the patients with positive p53 expression. The OS (**A**) and PFS (**B**) were compared among the patients treated with Auto-SCT or Allo-SCT or without HSCT

The five-year OS and PFS rates were 64% (95% CI 59–68) and 37% (95% CI 32–42), respectively, for the entire cohort of patients; 48% (95% CI 42–55) and 20% (95% CI 15–27), respectively, for the older cohort; and 78% (95% CI 73–83) and 51% (95% CI 45–58), respectively, for the younger cohort (Fig. 1). Of the 551 cases with complete treatment history, 512 patients received treatment and 39 were managed with “watch and wait.” R-CHOP and R-hyperCVAD were the two most common front-line regimens, accounting for 45% and 30% of chemotherapy-treated patients, respectively. We categorized the cases into four groups based on the chemotherapy regimens: anthracycline-based, cytarabine-based, purine analogue-based, and others. Notably, in the entire cohort analysis, patients treated with cytarabine-based regimens demonstrated superior outcomes compared to those receiving anthracycline-based regimens (*p* = 0.0028 for OS and *p* < 0.0001 for PFS) (Additional file 1: Fig. S2). However, this likely reflects selection bias, as 53% of the patients treated with cytarabine-based chemotherapy also received HSCT, compared to only 27% of those on non-cytarabine-based regimens. Indeed, when we incorporated HSCT into our assessment, the perceived advantage of cytarabine-based chemotherapy disappeared for the OS (Fig. 2). Nevertheless, cytarabine-based chemotherapy retained its efficacy in delaying disease progression, particularly when combined with HSCT, leading to a significant improvement in PFS. This benefit was most noticeable in younger patients, who are likely more tolerant of the treatment (Fig. 2). HSCT was carried out in 33% of the patients, of which 80% received autologous stem cell transplantation (auto-SCT) and 20% received allogeneic stem cell transplantation (allo-SCT). HSCT was the most significant treatment parameter affecting outcomes in all the analyzed cohorts (entire cohort, *p* < 0.0001 for both OS and PFS; older cohort, *p* = 0.0005 for OS, and *p* = 0.0018 for PFS; younger cohort, *p* = 0.0015 for OS, and *p* = 0.0003 for PFS, Additional file 1: Table S1 and Fig. S3), as well as for the blastoid/pleomorphic variant (*p* = 0.01 for OS and *p* = 0.0114 for PFS, Additional file 1: Fig. S4). In addition, we analyzed the impact of HSCT on the patients with positive p53 expression and found that those treated with auto-SCT exhibited superior outcomes, especially when compared to those not receiving HSCT (*p* = 0.008 for OS, and *p* = 0.024 for PFS, Fig. 3). Rituximab maintenance was administered in 20% of the patients, leading to improved PFS and OS in both the entire cohort and the older cohort, whereas no significant improvement was observed in the younger cohort (Additional file 1: Table S1).

### Univariate analysis of baseline characteristics

Univariate analysis was conducted on various baseline parameters, with the results listed in Additional file 2: Table S2 and summarized in Additional file 1: Table S1. For the clinical and laboratory parameters, age, ECOG performance (≥ 2), B symptoms, and anemia were the parameters most significantly associated with both OS and PFS, in the entire cohort. Other factors, including spleen involvement, thrombocytopenia, CTCs, Ann Arbor stage (AAS) ≥ III, higher LDH, and multifocal disease were also significantly correlated with inferior outcomes, whereas colon involvement was correlated with better OS. Analysis based on age-defined groups revealed that ECOG performance and anemia were highly significant in the older cohort, whereas B symptoms emerged as the most significant parameter in the younger cohort. Furthermore, age, when examined as a continuous variable, exhibited a strong correlation with outcomes in both the entire cohort and the older cohort. However, this correlation was not significant within the younger cohort.

All the pathological parameters, except for CD5 expression, had prognostic significance, with p53 IHC-positive being the most notable one (Additional file 1: Fig. S5). Specifically, in the entire cohort, positive p53, high Ki-67, diffuse growth pattern, and SOX11 were significantly correlated with inferior outcomes for both OS and PFS, whereas ILCR, morphological variants, and CD23 had significance only for the OS. In the older cohort, diffuse growth pattern and p53 positivity retained the prognostic value for both OS and PFS, while the significance of others was lost or largely decreased. In the younger cohort, p53, CD23, Ki-67, SOX11, and morphological variants had prognostic significance for OS, whereas none of the pathological parameters predicted PFS.

### MIPI and MIPI-c stratification

The MIPI and MIPI-c scores were obtained in 109 patients. In MIPI stratification, 11% of the cases were classified as high risk, 52% as intermediate risk, and 37% as low risk. In MIPI-c stratification, 11% of the cases were classified as high risk, 29% as high intermediate risk, 27% as low intermediate risk, and 33% as low risk. Both the MIPI and MIPI-c demonstrated significant stratification of the groups (p < 0.0001), excelling particularly in identifying the low-risk group. However, they were less effective in differentiating between the intermediate- and high-risk groups (Additional file 1: Fig. S6A, B). Furthermore, when evaluating the PFS of groups divided by the MIPI and MIPI-c, the stratification was found to be much less effective (Additional file 1: Fig. S6C, D).

### Multivariate analysis and prognostic regression model

**Fig. 4:**
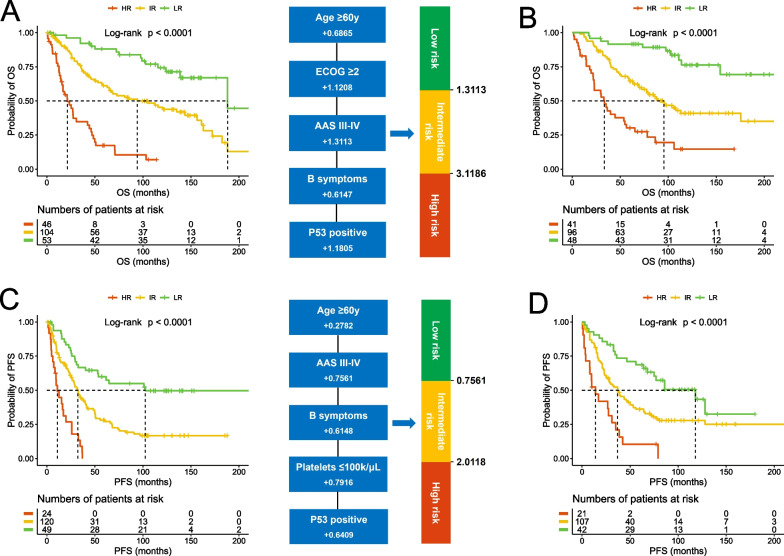
Development of new overall prognostic models. The OS prognostic model was developed in the training cohort (**A**) and tested in the validation cohort (**B**). The PFS prognostic model was developed in the training cohort (**C**) and tested in the validation cohort (**D**)

To develop new prognostic models, multivariate analysis and Cox regression were performed for OS and PFS, respectively. For OS, a total of 203 cases passed the screening for data completeness and were subsequently employed for the evaluation. In this refined cohort, p53 immunostaining emerged as the most significant variable in the Cox regression with backward variable selection, followed by ECOG performance, age (≥ 60y as a more effective cutoff than ≥ 65y), B symptoms, and AAS. Accordingly, the optimal prognostic index was developed as 0.6865 (if age ≥ 60y) + 1.1208 (if ECOG ≥ 2) + 1.3113 (if AAS III-IV) + 0.6147 (if having B symptoms) + 1.1805 (if p53 positive). By this calculation, the cases were classified into low-risk (26.1%, score ≤ 1.3113), intermediate-risk (51.2%, 1.3113 < score ≤ 3.1186), and high-risk (22.7%, score > 3.1186) groups with distinctive OS rates (*p* < 0.0001, Fig. 4A). We then tested this model in an independent validation cohort of 185 cases, which exhibited similar survival rates to the training cohort (Additional file 1: Fig. S7). Notably, the treatment approaches in the validation cohort were more reflective of contemporary medical care, with 41% of the cases being treated with Bendamustine and 23% receiving BTK inhibitor treatment. Despite the difference in treatment approach, the prognostic model resulted in highly consistent stratification with the training cohort, especially regarding the percentage distribution for each group (25.9%, 51.9%, and 22.2% for low, intermediate, and high risk, respectively, Fig. 4B). For PFS, the analysis was successfully carried out in 193 cases, and B symptoms, p53, platelet count (≤ 100 k/μL), AAS, and age were selected by the backward variable selection. Accordingly, the prognostic index was calculated as 0.2782 (if age ≥ 60y) + 0.7561 (if AAS III-IV) + 0.6148 (if having B symptoms) + 0.7916 (if platelets ≤ 100 k/µL) + 0.6409 (if p53 positive). By this calculation, the cohort was classified into low-risk (25.4%, score ≤ 0.7561), intermediate-risk (62.2%, 0.7561 < score ≤ 2.0118), and high-risk (12.4%, score > 2.0118) groups with distinctive PFS rates (*p* < 0.0001, Fig. 4C). Also, this model demonstrated strong predictive power and high consistency in the validation cohort (24.7%, 62.9%, and 12.4% for low-, intermediate- and high-risk groups, respectively, Fig. 4D).

**Fig. 5:**
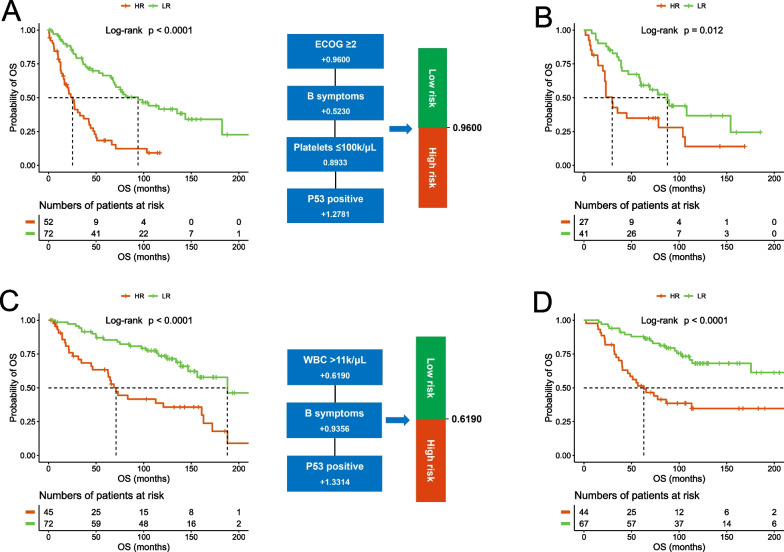
Development of new age-specific prognostic models. The prognostic model for older patients (≥ 65y) was developed in the training cohort (**A**) and tested in the validation cohort (**B**); the prognostic model for younger patients (< 65y) was developed in the training cohort (**C**) and tested in the validation cohort (**D**)

These two new prognostic models, like the currently used models, also include patient age in the algorithms. However, this becomes an issue as more and more practices, especially emerging clinical trials, are conducted in an age-defined manner. To meet these needs, we also developed two age-specific prognostic models with a cutoff age of 65y, using the same training and validation methods. Specifically, for the older cohort (≥ 65y), the prognostic index was calculated as 0.9600 (if ECOG ≥ 2) + 0.5230 (if having B symptoms) + 0.8933 (if platelets ≤ 100 k/µL) + 1.2781 (if p53 positive) with the score ≤ 0.9600 for the low-risk group (58.1% and 60.3% for training and validation, respectively), and > 0.9600 for the high-risk group (41.9% and 39.7%) (Fig. 5A, B). For the younger cohort (< 65y), the prognostic index was calculated as 0.6190 (if WBC > 11 k/μL) + 0.9356 (if having B symptoms) + 1.3314 (if p53 positive) with the score ≤ 0.6190 for the low-risk group (61.5% and 60.4%), and > 0.6190 for the high-risk group (38.5% and 39.6%) (Fig. 5C, D).

To distinguish this stratification method from the existing models, we named it the North American MCL Prognostic Indexes (NAMPIs), which can be accessed on the following platform: https://unmcredcap.unmc.edu/redcap/surveys/?s=WNFCJWACLMDC9WKN

## Discussion

The current study retrospectively investigated a large cohort of MCL cases treated in the immunochemotherapy era. It revealed survival rates superior to those of previous cohorts treated only with chemotherapy [15, 16], suggesting that the broad use of rituximab has indeed improved the overall outcome of MCL patients in the real world. Moreover, HSCT conferred significant clinical benefits by remarkably improving outcomes compared to immunochemotherapy alone and appears to be an effective approach for managing the blastoid/pleomorphic variant. In terms of chemotherapy strategies, cytarabine-based regimens with HSCT significantly improved PFS in younger patients. This is in accordance with previous studies recommending cytarabine-based regimens followed by HSCT as a preference for eligible young patients [17, 18]. Although no significant advantages have been shown for OS, which indicates a similar proportion of relapses among these patients compared to those treated with non-cytarabine-based induction, there remains a discernible trend favoring cytarabine-based regimens and HSCT as initial therapy. Meanwhile, this observation underscores the need for improved management strategies to further enhance the long-term outcomes of this approach.

Despite these advances, MCL remains an incurable disease for most patients. Within the current therapeutic landscape, several options are available for front-line treatment. Additionally, the integration of approved second-line agents, such as BTK inhibitors, bortezomib, and lenalidomide into front-line therapy holds promise for improving disease management. The selection of the most suitable approach relies on the accurate risk evaluation of patients. Our study aimed to establish new risk stratification models incorporating the major clinicopathological features that would effectively inform current clinical practice. Our univariate analysis recapitulates the risk factor profile of the clinical and laboratory parameters [4, 6, 19], except for the new findings that thrombocytopenia and CTCs were correlated with inferior outcomes, whereas colon involvement was correlated with superior outcomes. It is worth noting that CTCs also occur in leukemic non-nodal MCL, which is a recently defined indolent variant with slow or absent clinical progression. As this variant was not established at the diagnosis of this cohort of cases, to address this issue, we analyzed the CTCs in only the cases with nodal involvement and found it to have similar prognostic significance (Additional file 2: Table S2). As for colon involvement, there might be a bias in patient inclusion as patients with aggressive MCL or poor general conditions usually are not subject to colon biopsy. Indeed, colon involvement did not significantly impact the outcomes of the younger cohort, in which colonoscopy was more likely to be performed. Therefore, additional studies are needed to specifically address this issue.

For the pathological features, in addition to the Ki-67 index, we were particularly interested in the p53 status because studies have demonstrated that p53 is frequently mutated in MCL, and is associated with treatment resistance and inferior outcomes [20–23]. Moreover, p53 expression measured by IHC staining has high sensitivity and specificity in predicting the missense mutations and serves as a potential biomarker to predict clinical outcomes, even independent of the MIPI and Ki-67 [21, 24]. In this study, instead of simply using the percentage of positive cells, we employed a semi-quantitative scoring method to determine the p53 expression level. In fact, this method had a high concordance (> 90%) with the estimation by percentage using 30% as the cutoff in our study. This method also effectively minimized the discrepancies among pathologists in cases exhibiting a high percentage of weak positivity. Strikingly, we found that p53 expression was a stronger predictor of outcome than Ki-67. Moreover, during all risk models’ development, only the p53 expression was consistently selected by Cox regression. This underscores its significance in treatment response and outcome, especially in the context of integrative analysis of the baseline parameters. A limitation of the current study is the sole involvement of p53 expression without considering the genetic status of *TP53*. Previous studies have indicated that discrepancies between expression and mutation can be approximately 10–20% [21, 25], with genetic sequencing recommended as the more accurate method for determining p53 status. However, given the complexity of p53 alterations in MCL, protein expression holds significant value due to its functional relevance. This is emphasized by mounting evidence highlighting a strong correlation between p53 expression and prognosis [26, 27]. Additionally, detecting expression by IHC is a more accessible method for routine clinical practice, making it especially valuable for low- to medium-income areas.

In evaluating other pathological features, the diffuse growth pattern stands out as being notably correlated with inferior outcomes for both OS and PFS in the entire cohort as well as in older patients. The blastoid/pleomorphic cytology, ILCR, and CD23 positivity also demonstrated prognostic value in relation to inferior outcomes, whereas CD5 showed no significance across all analyzed cohorts. Among these, the blastoid/pleomorphic cytology was reproducibly determined as a risk factor in previous studies [5, 28, 29]. Notably, our findings reveal that HSCT significantly improves the survival rates for patients with the blastoid/pleomorphic variant, suggesting that this treatment should be considered a priority for these patients. Interestingly, while CD23 was identified as a biomarker associated with better outcomes in previous studies [30, 31], it demonstrated a negative correlation with survival in our study. This discrepancy might be attributed to the low proportion of CD23 positivity in MCL. None of these parameters were selected by the Cox regression for risk model development, implying a potential overlap with other features.

Unlike other prognostic models, our NAMPIs include a PFS model to better assist with clinical decisions. A potential application scheme could be as follows: for the patients stratified as low-risk by the PFS model, less aggressive regimens, such as bendamustine/rituximab, are preferred. In contrast, for high-risk patients, especially those identified by both OS and PFS models, HSCT, clinical trials, and rituximab maintenance are highly recommended for consideration. Additionally, early intervention with BTK inhibitors, potentially as part of front-line therapy, is advised for these patients to optimize outcomes, as this is a cutting-edge trend for MCL treatment. Two recent extensive cohort studies have underscored the potential of this strategy [23, 32]. For intermediate-risk patients, a balanced approach is recommended, carefully weighing the potential benefits and risks of both standard and more aggressive treatments while taking into consideration other healthcare issues. Furthermore, we have also developed two models specifically tailored for older and younger patients. This initiative was aimed at fulfilling the need for new therapy development, as there is an increasing trend of investigations conducted specifically in younger or older patient populations. All of the established models were validated in an independent cohort of cases. Importantly, a higher percentage of patients in the validation cohort received R-bendamustine or BTK inhibitor treatment. Despite this, the stratification closely mirrored the training cohort, suggesting that these models are effective across various treatment approaches and would serve as valuable tools to improve the clinical management of MCL patients. While the NAMPI models are anchored in clinical and pathological data, integrating prognosticators from a deeper molecular level, such as MCL35 [33], could enable a more nuanced and multidimensional patient stratification. Such integration may further enhance our ability to tailor treatments to the specific needs of each patient, potentially improving outcomes in MCL management.

In conclusion, through a multi-institutional collaboration in North America, we analyzed the risk factors associated with outcomes in the immunochemotherapy era for MCL and developed novel risk stratification models for both general and age-specific applications. Future studies are needed to incorporate genetic features into risk stratification, especially for the purpose of improving the administration of second-line and novel therapies.

### Supplementary Information

**Additional file 1**. Supplementary Table S1 and Supplementary Figures.

**Additional file 2**. Supplementary Table S2.

## Abbreviations

AAS: Ann Arbor stage
Allo-SCT: Allogeneic stem cell transplantation
Auto-SCT: Autologous stem cell transplantation
BTK: Bruton tyrosine kinase
CAR-T: Chimeric antigen receptor T cell
CTC: Circulating tumor cell
ECOG: Eastern Cooperative Oncology Group
HSCT: Hematopoietic stem cell transplantation
IHC: Immunohistochemistry
ILCR: Immunoglobulin light chain restriction
LDH: Lactate dehydrogenase
MCL: Mantle cell lymphoma
MIPI: MCL International Prognostic Index
MIPI-c: Combined MCL International Prognostic Index
NAMPIs: North American MCL Prognostic Indexes
NAMCLP: North American Mantle Cell Lymphoma Project
OS: Overall survival
PFS: Progression-free survival
UNMC: The University of Nebraska Medical Center

## References

[1] Vose JM (2017). Mantle cell lymphoma: 2017 update on diagnosis, risk-stratification, and clinical management. Am J Hematol.

[2] Jeon YW, Yoon S, Min GJ, Park SS, Park S, Yoon JH (2019). Clinical outcomes for ibrutinib in relapsed or refractory mantle cell lymphoma in real-world experience. Cancer Med.

[3] Epperla N, Hamadani M, Fenske TS, Costa LJ (2018). Incidence and survival trends in mantle cell lymphoma. Br J Haematol.

[4] Hoster E, Dreyling M, Klapper W, Gisselbrecht C, van Hoof A, Kluin-Nelemans HC (2008). A new prognostic index (MIPI) for patients with advanced-stage mantle cell lymphoma. Blood.

[5] Hoster E, Rosenwald A, Berger F, Bernd HW, Hartmann S, Loddenkemper C (2016). Prognostic value of Ki-67 Index, cytology, and growth pattern in mantle-cell lymphoma: results from randomized trials of the European mantle cell lymphoma network. J Clin Oncol.

[6] Schaffel R, Hedvat CV, Teruya-Feldstein J, Persky D, Maragulia J, Lin D (2010). Prognostic impact of proliferative index determined by quantitative image analysis and the International Prognostic Index in patients with mantle cell lymphoma. Ann Oncol.

[7] Salek D, Vesela P, Boudova L, Janikova A, Klener P, Vokurka S (2014). Retrospective analysis of 235 unselected patients with mantle cell lymphoma confirms prognostic relevance of Mantle Cell Lymphoma International Prognostic Index and Ki-67 in the era of rituximab: long-term data from the Czech Lymphoma Project Database. Leuk Lymphoma.

[8] Hoster E, Klapper W, Hermine O, Kluin-Nelemans HC, Walewski J, van Hoof A (2014). Confirmation of the mantle-cell lymphoma International Prognostic Index in randomized trials of the European Mantle-Cell Lymphoma Network. J Clin Oncol.

[9] Determann O, Hoster E, Ott G, Wolfram Bernd H, Loddenkemper C, Leo Hansmann M (2008). Ki-67 predicts outcome in advanced-stage mantle cell lymphoma patients treated with anti-CD20 immunochemotherapy: results from randomized trials of the European MCL Network and the German Low Grade Lymphoma Study Group. Blood.

[10] McGraw KL, Nguyen J, Komrokji RS, Sallman D, Al Ali NH, Padron E (2016). Immunohistochemical pattern of p53 is a measure of TP53 mutation burden and adverse clinical outcome in myelodysplastic syndromes and secondary acute myeloid leukemia. Haematologica.

[11] Schoenfeld D (1982). Partial residuals for the proportional hazards regression model. Biometrika.

[12] Grambsch P, Therneau T (1994). Proportional hazards tests and diagnostics based on weighted residuals. Biometrika.

[13] Altman DG, Lausen B, Sauerbrei W, Schumacher M (1994). Dangers of using "optimal" cutpoints in the evaluation of prognostic factors. J Natl Cancer Inst.

[14] Efron B, Tibshirani R (1994). An introduction to the bootstrap.

[15] Lenz G, Dreyling M, Hoster E, Wormann B, Duhrsen U, Metzner B (2005). Immunochemotherapy with rituximab and cyclophosphamide, doxorubicin, vincristine, and prednisone significantly improves response and time to treatment failure, but not long-term outcome in patients with previously untreated mantle cell lymphoma: results of a prospective randomized trial of the German Low Grade Lymphoma Study Group (GLSG). J Clin Oncol.

[16] Nickenig C, Dreyling M, Hoster E, Pfreundschuh M, Trumper L, Reiser M (2006). Combined cyclophosphamide, vincristine, doxorubicin, and prednisone (CHOP) improves response rates but not survival and has lower hematologic toxicity compared with combined mitoxantrone, chlorambucil, and prednisone (MCP) in follicular and mantle cell lymphomas: results of a prospective randomized trial of the German Low-Grade Lymphoma Study Group. Cancer.

[17] Hermine O, Hoster E, Walewski J, Bosly A, Stilgenbauer S, Thieblemont C (2016). Addition of high-dose cytarabine to immunochemotherapy before autologous stem-cell transplantation in patients aged 65 years or younger with mantle cell lymphoma (MCL Younger): a randomised, open-label, phase 3 trial of the European Mantle Cell Lymphoma Network. Lancet.

[18] Merryman RW, Edwin N, Redd R, Bsat J, Chase M, LaCasce A (2020). Rituximab/bendamustine and rituximab/cytarabine induction therapy for transplant-eligible mantle cell lymphoma. Blood Adv.

[19] Samaha H, Dumontet C, Ketterer N, Moullet I, Thieblemont C, Bouafia F (1998). Mantle cell lymphoma: a retrospective study of 121 cases. Leukemia.

[20] Eskelund CW, Dahl C, Hansen JW, Westman M, Kolstad A, Pedersen LB (2017). TP53 mutations identify younger mantle cell lymphoma patients who do not benefit from intensive chemoimmunotherapy. Blood.

[21] Rodrigues JM, Hassan M, Freiburghaus C, Eskelund CW, Geisler C, Raty R (2020). p53 is associated with high-risk and pinpoints TP53 missense mutations in mantle cell lymphoma. Br J Haematol.

[22] Nadeu F, Martin-Garcia D, Clot G, Diaz-Navarro A, Duran-Ferrer M, Navarro A (2020). Genomic and epigenomic insights into the origin, pathogenesis, and clinical behavior of mantle cell lymphoma subtypes. Blood.

[23] Wang ML, Jurczak W, Jerkeman M, Trotman J, Zinzani PL, Belada D (2022). Ibrutinib plus bendamustine and rituximab in untreated mantle-cell lymphoma. N Engl J Med.

[24] Aukema SM, Hoster E, Rosenwald A, Canoni D, Delfau-Larue MH, Rymkiewicz G (2018). Expression of TP53 is associated with the outcome of MCL independent of MIPI and Ki-67 in trials of the European MCL Network. Blood.

[25] Nolan J, Murphy C, Dinneen K, Lee G, Higgins E, Bacon L (2022). p53 immunohistochemistry must be confirmed by TP53 next generation sequencing for accurate risk stratification of patients with mantle cell lymphoma. Leuk Lymphoma.

[26] Xie Y, Bulbul MA, Ji L, Inouye CM, Groshen SG, Tulpule A (2014). p53 expression is a strong marker of inferior survival in de novo diffuse large B-cell lymphoma and may have enhanced negative effect with MYC coexpression: a single institutional clinicopathologic study. Am J Clin Pathol.

[27] Scheubeck G, Jiang L, Hermine O, Kluin-Nelemans HC, Schmidt C, Unterhalt M, Rosenwald A, Klapper W, Evangelista A, Ladetto M, Jerkeman M (2023). Clinical outcome of Mantle Cell Lymphoma patients with high-risk disease (high-risk MIPI-c or high p53 expression). Leukemia.

[28] Bernard M, Gressin R, Lefrere F, Drenou B, Branger B, Caulet-Maugendre S (2001). Blastic variant of mantle cell lymphoma: a rare but highly aggressive subtype. Leukemia.

[29] Dreyling M, Klapper W, Rule S (2018). Blastoid and pleomorphic mantle cell lymphoma: still a diagnostic and therapeutic challenge!. Blood.

[30] Kelemen K, Peterson LC, Helenowski I, Goolsby CL, Jovanovic B, Miyata S (2008). CD23+ mantle cell lymphoma: a clinical pathologic entity associated with superior outcome compared with CD23- disease. Am J Clin Pathol.

[31] Hu Z, Sun Y, Schlette EJ, Tang G, Li S, Xu J (2018). CD200 expression in mantle cell lymphoma identifies a unique subgroup of patients with frequent IGHV mutations, absence of SOX11 expression, and an indolent clinical course. Mod Pathol.

[32] Dreyling M, Doorduijn JK, Gine E, Jerkeman M, Walewski J, Hutchings M (2022). Efficacy and safety of ibrutinib combined with standard first-line treatment or as substitute for autologous stem cell transplantation in younger patients with mantle cell lymphoma: results from the randomized triangle trial by the European MCL network. Blood.

[33] Holte H, Beiske K, Boyle M, Troen G, Blaker YN, Myklebust J (2018). The MCL35 gene expression proliferation assay predicts high-risk MCL patients in a Norwegian cohort of younger patients given intensive first line therapy. Br J Haematol.

