# Supplementary material for: Integrative analysis of clinicopathological features defines novel prognostic models for mantle cell lymphoma in the immunochemotherapy era: a report from The North American Mantle Cell Lymphoma Consortium
Source: J Hematol Oncol. 2023 Dec 16;16:122. doi: 10.1186/s13045-023-01520-7 (PMC10725579; doi:10.1186/s13045-023-01520-7)

Supplementary Table S1. Summary of Univariate Analysis

Parameters	*P value*		Parameters	*P value*		Parameters	*P value*	
	OS	PFS		OS	PFS		OS	PFS
			**AAS**	**0.0016**	**0.00072**	**Clonal Serum Ig**	**0.148**	**0.0298**
**Age**	**1.57E-15**	**1.32E-10**	Older Cohort	0.330	0.154	Older Cohort	0.925	0.120
			Younger Cohort	0.0116	0.00778	Younger Cohort	0.261	0.647
**Gender**	**0.395**	**0.335**	**Extranodal site involved**	**0.390**	**0.525**	**WBCs**	**0.000201**	**0.287**
Older Cohort	0.482	0.224	Older Cohort	0.0944	0.330	Older Cohort	0.0286	0.806
Younger Cohort	0.462	0.567	Younger Cohort	0.966	0.521	Younger Cohort	0.00334	0.501
**Race**	**0.466**	**0.831**	**Limited to one site**	**0.00366**	**0.0102**	**Neutrophils**	**0.0147**	**0.0426**
Older Cohort	0.711	0.676	Older Cohort	0.220	0.534	Older Cohort	0.0958	0.109
Younger Cohort	0.437	0.630	Younger Cohort	0.0277	0.0209	Younger Cohort	0.315	0.491
**Performance**	**1.30E-10**	**1.23E-05**	**Circulating tumor cells**	**4.89E-05**	**0.00915**	**Lymphocytes**	**0.00431**	**0.957**
Older Cohort	1.47E-06	0.00325	Older Cohort	0.00683	0.0817	Older Cohort	0.192	0.798
Younger Cohort	0.0189	0.0425	Younger Cohort	0.00132	0.122	Younger Cohort	0.00666	0.712
**Tonsil involved**	**0.114**	**0.733**	**Largest diameter**	**0.0367**	**0.677**	**Anemia**	**2.69E-06**	**2.14E-05**
Older Cohort	0.762	0.906	Older Cohort	0.735	0.900	Older Cohort	0.00128	0.0363
Younger Cohort	0.200	0.883	Younger Cohort	0.0169	0.623	Younger Cohort	0.00364	0.00138
**Colon involved**	**0.00788**	**0.711**	**R maintenance**	**0.0045**	**0.00832**	**Platelets**	**3.16E-05**	**0.00225**
Older Cohort	0.0993	0.240	Older Cohort	0.000313	0.00352	Older Cohort	0.00258	0.0215
Younger Cohort	0.0554	0.476	Younger Cohort	0.625	0.436	Younger Cohort	0.00166	0.0765

**Liver involved**	**0.0606**	**0.00296**	**Anthracycline-based vs Cytarabine-based**	**0.002823**	**1.44E-05**	**CD23**	**0.0414**	**0.881**
Older Cohort	0.206	0.00964	Older Cohort	0.345	0.44615	**Older Cohort**	**0.540**	**0.309**
Younger Cohort	0.022	0.00764	Younger Cohort	0.254	0.000297	**Younger Cohort**	**0.00304**	**0.314**
**Small intestine involved**	**0.436**	**0.377**	**HSCT**	**4.75E-12**	**5.03E-11**	**Diffuse growth pattern**	**0.000755**	**0.00343**
Older Cohort	0.532	0.325	Older Cohort	0.000485	0.00179	Older Cohort	0.000937	0.00486
Younger Cohort	0.896	0.973	Younger Cohort	0.00152	0.000337	Younger Cohort	0.478	0.242
**Spleen involved**	**1.46E-06**	**0.0143**	**Auto vs Allo HSCT**	**0.064**	**0.0196**	**Cytology**	**0.00864**	**0.0918**
Older Cohort	0.0176	0.452	Older Cohort	0.193	0.592	Older Cohort	0.425	0.137
Younger Cohort	2.76E-05	0.0567	Younger Cohort	0.0895	0.0182	Younger Cohort	0.0175	0.966
**Stomach involved**	**0.366**	**0.535**	**Elevated LDH**	**2.19E-05**	**0.0243**	**P53 expression**	**1.84E-09**	**0.00214**
Older Cohort	0.767	0.481	Older Cohort	0.0119	0.110	Older Cohort	8.49E-06	0.00713
Younger Cohort	0.451	0.940	Younger Cohort	0.000636	0.213	Younger Cohort	0.000706	0.178
**Digestive tract involved**	**0.0167**	**0.308**	**Elevated Beta-MG**	**0.00877**	**0.0714**	**Ki67**	**8.36E-05**	**0.00625**
Older Cohort	0.118	0.142	Older Cohort	0.264	0.636	Older Cohort	0.0237	0.0305
Younger Cohort	0.118	0.936	Younger Cohort	0.0094	0.0881	Younger Cohort	0.00414	0.170
**B Symptoms**	**2.40E-07**	**3.99E-07**	**CD5**	**0.0518**	**0.955**	**SOX11**	**0.00262**	**0.0388**
Older Cohort	0.0228	0.140	Older Cohort	0.314	0.785	Older Cohort	0.135	0.129
Younger Cohort	9.12E-06	8.86E-07	Younger Cohort	0.0945	0.496	Younger Cohort	0.00762	0.148

Abbreviation: AAS, Ann Arbor stage, Beta-MG, beta-microglobulin; LDH, lactate dehydrogenase; SCT, stem cell transplantation; WBC, white blood cell.

Supplementary Figure Legends:

Supplementary Figure S1: The age distribution of the analytic cohort of MCL patients.

Supplementary Figure S2: Chemotherapy regimens and outcomes. The cases were divided into four groups according to chemotherapy regimens: anthracycline-based, cytarabine-based, purine analogue-based, and others. The OS and PFS of the entire cohort (A and B), the older cohort (C and D), and the younger cohort (E and F) are respectively shown.

Supplementary Figure S3: Impact of HSCT on survival. Comparison of the OS and PFS between patients treated with or without HSCT in the entire cohort (A and B), the older cohort (C and D), and the younger cohort (E and F).

Supplementary Figure S4: Impact of HSCT on the survival of the patients with blastoid/pleomorphic variant. Comparison of OS (A) and PFS (B) between patients with the blastoid/pleomorphic variant who received HSCT or not.

Supplementary Figure S5: The p53-IHC analysis in the MCL cases. (A-D) The various intensity of p53-IHC staining is shown: (A) negative staining; (B) weak and scattered intermediate positivity; (C) a mix of intermediate and strong positivity; and (D) strong positivity. The OS and PFS were compared between patients with positive and negative p53 in the entire cohort (E), the older cohort (F), and the younger cohort (G), respectively.

Supplementary Figure S6. The MIPI and MIPI-c stratification for the analytic cohort of cases. The OS (A and B), and PFS (C and B) analysis of the groups stratified by the MIPI and MIPI-c.

Supplementary Figure S7. Survival rates of the validation cohort. The OS and PFS are shown in overall (A and B) and age-defined manners (C and D).

Supplementary Figure S1

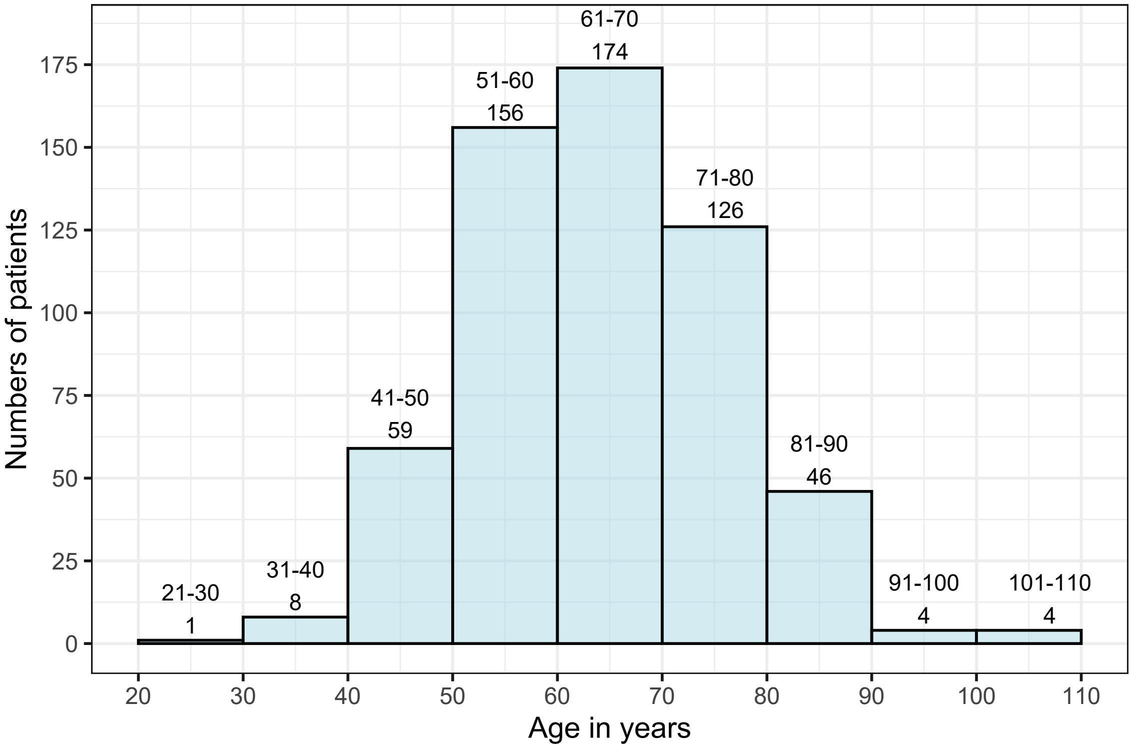

Supplementary Figure S2

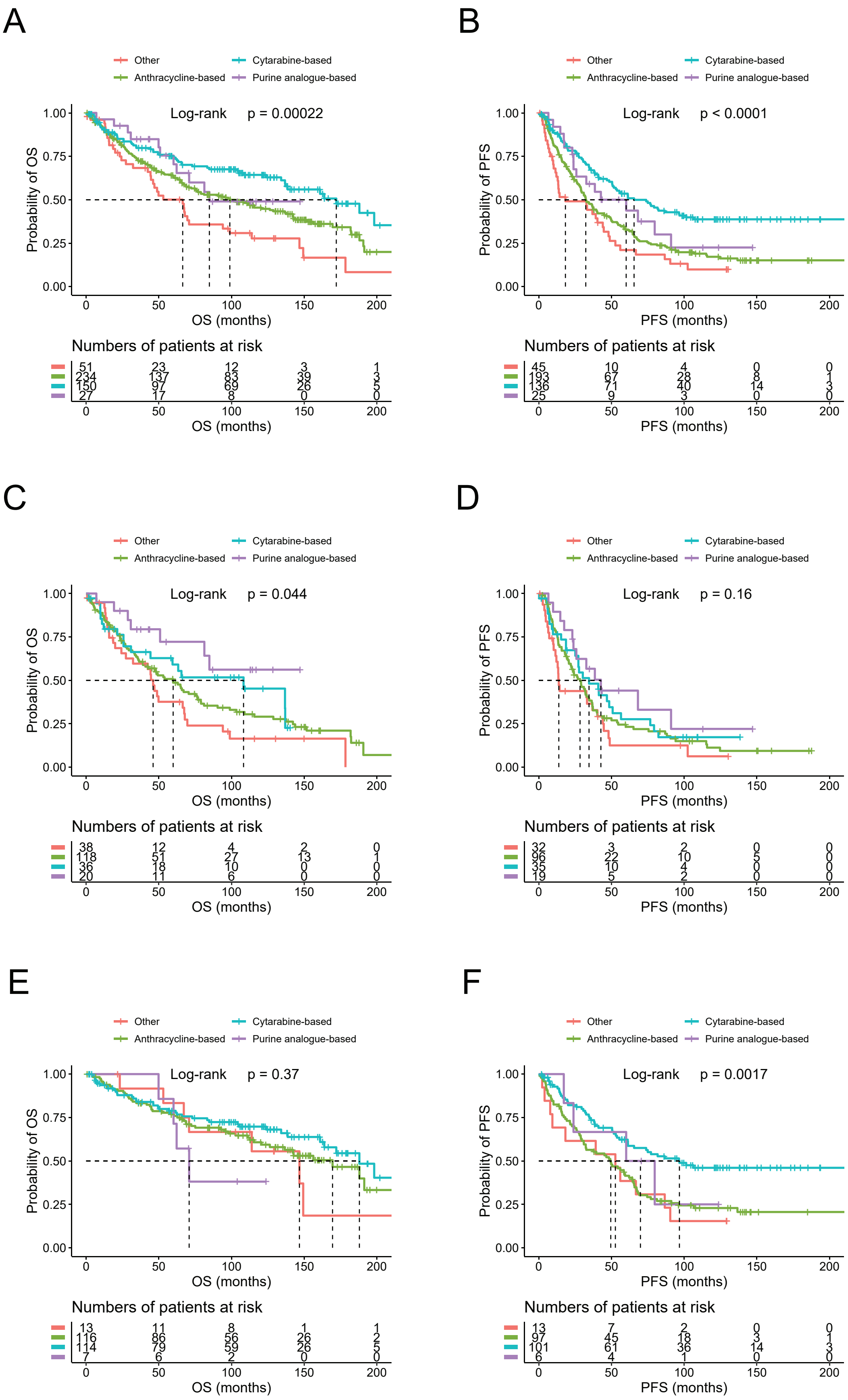

Supplementary Figure S3

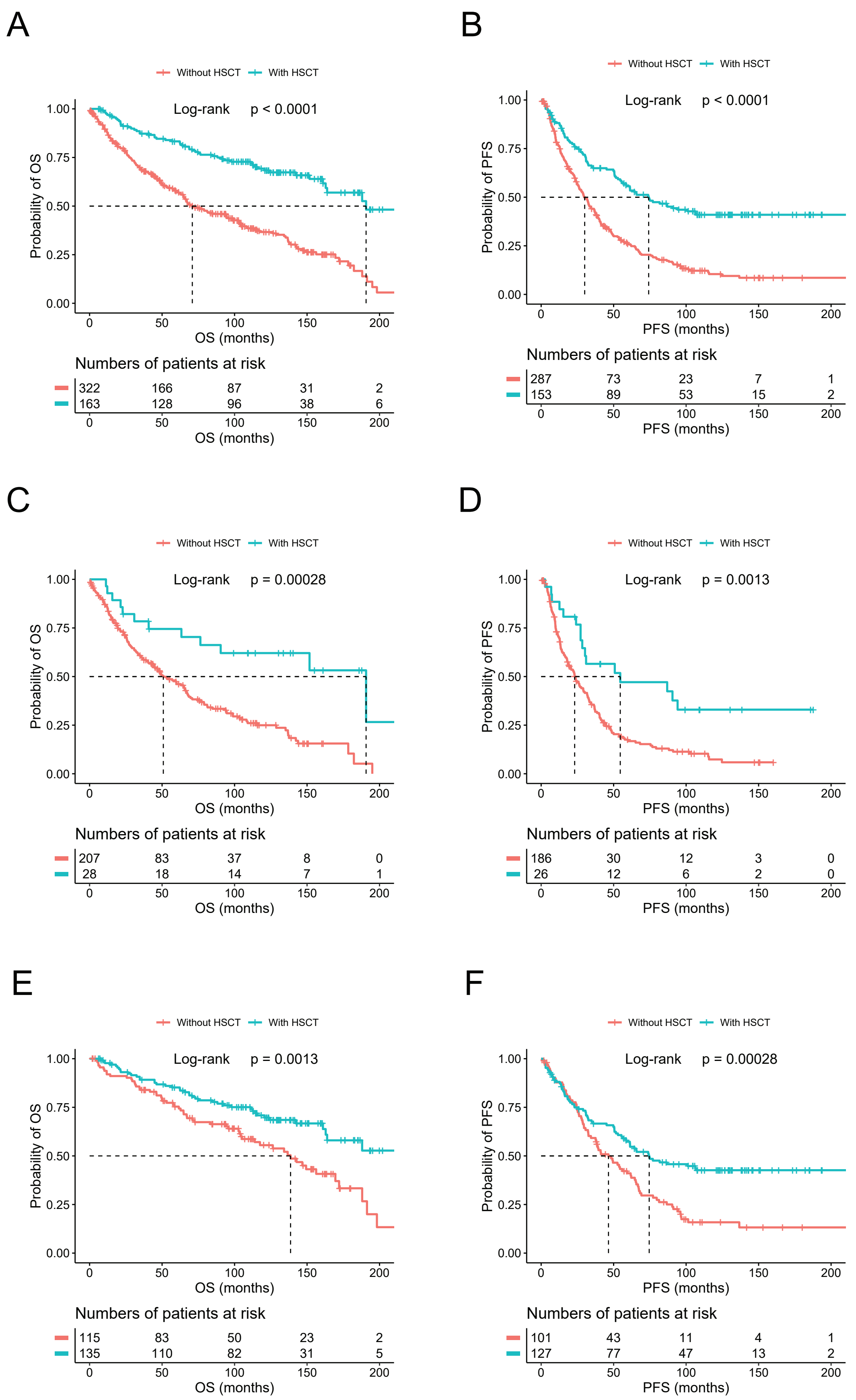

Supplementary Figure S4

A

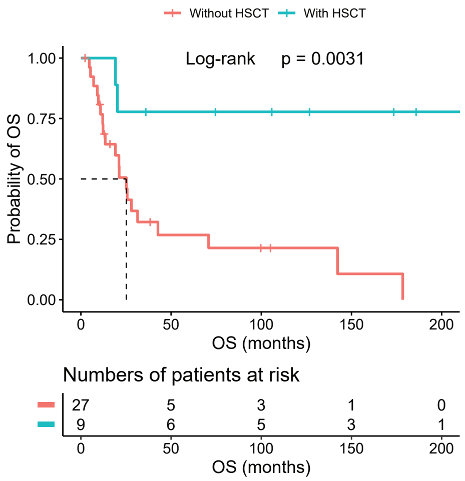

B

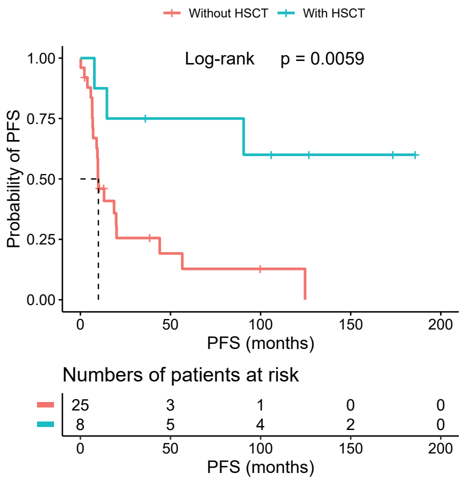

Supplementary Figure S5

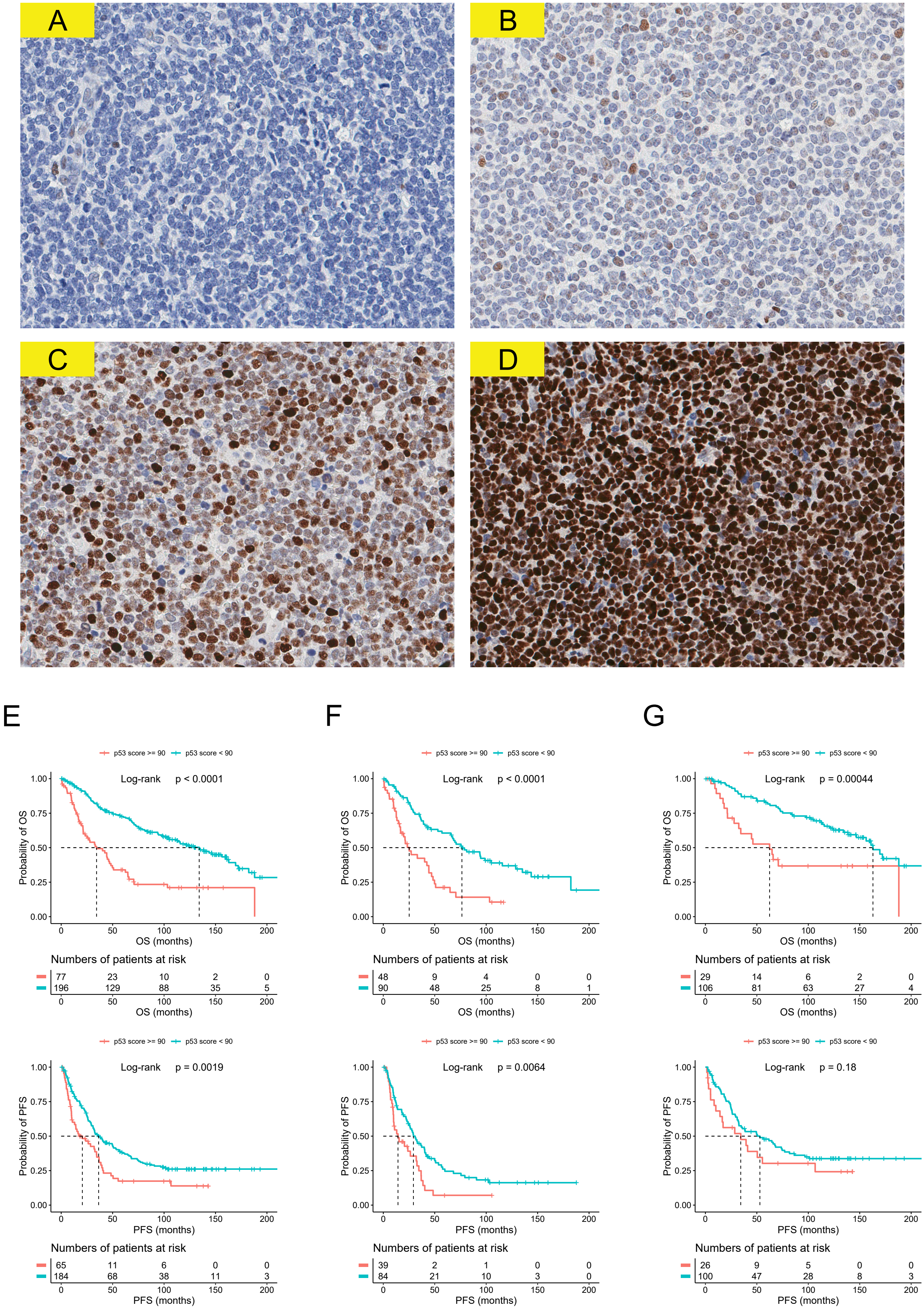

Supplementary Figure S6

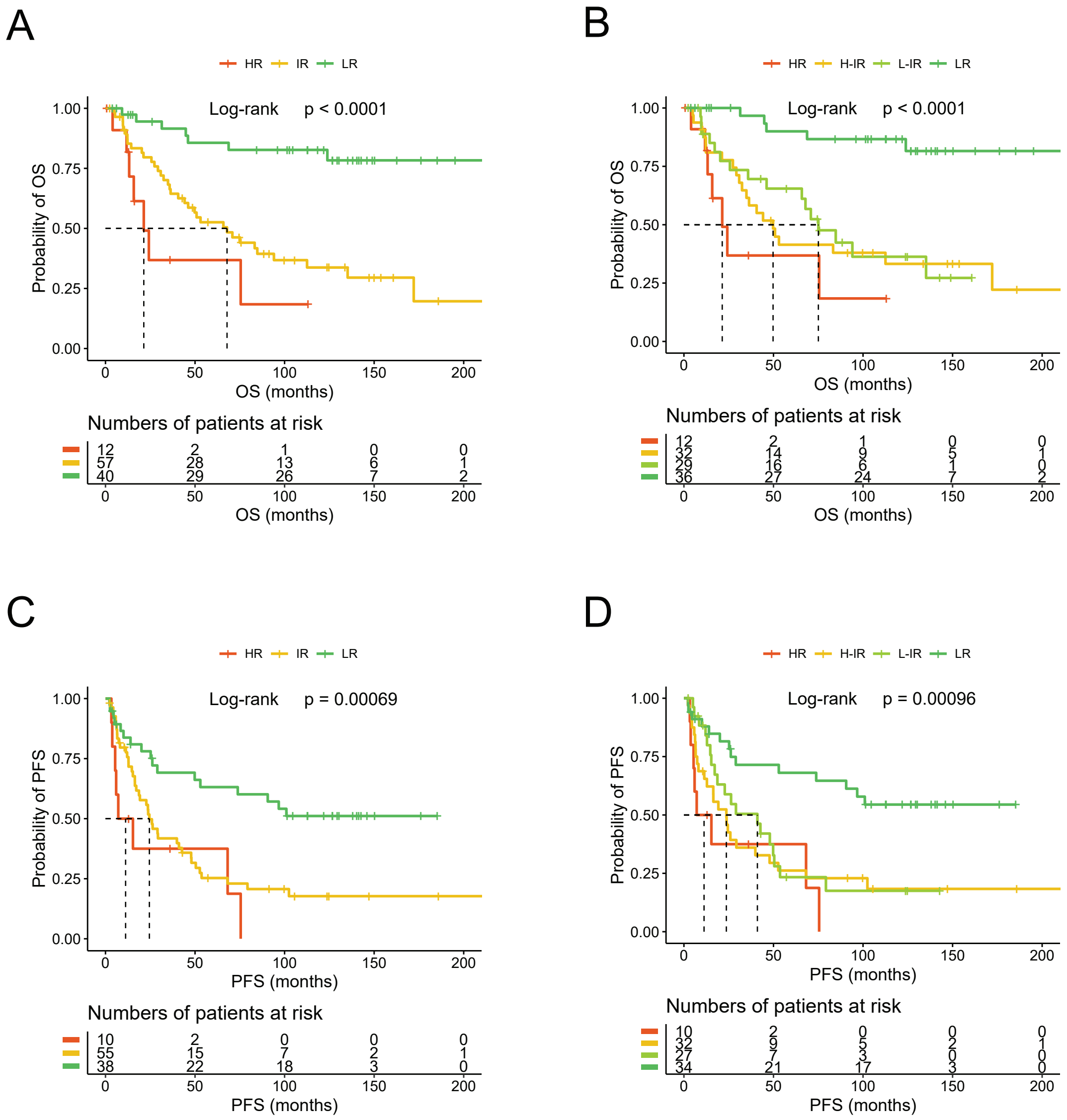

Supplementary Figure S7

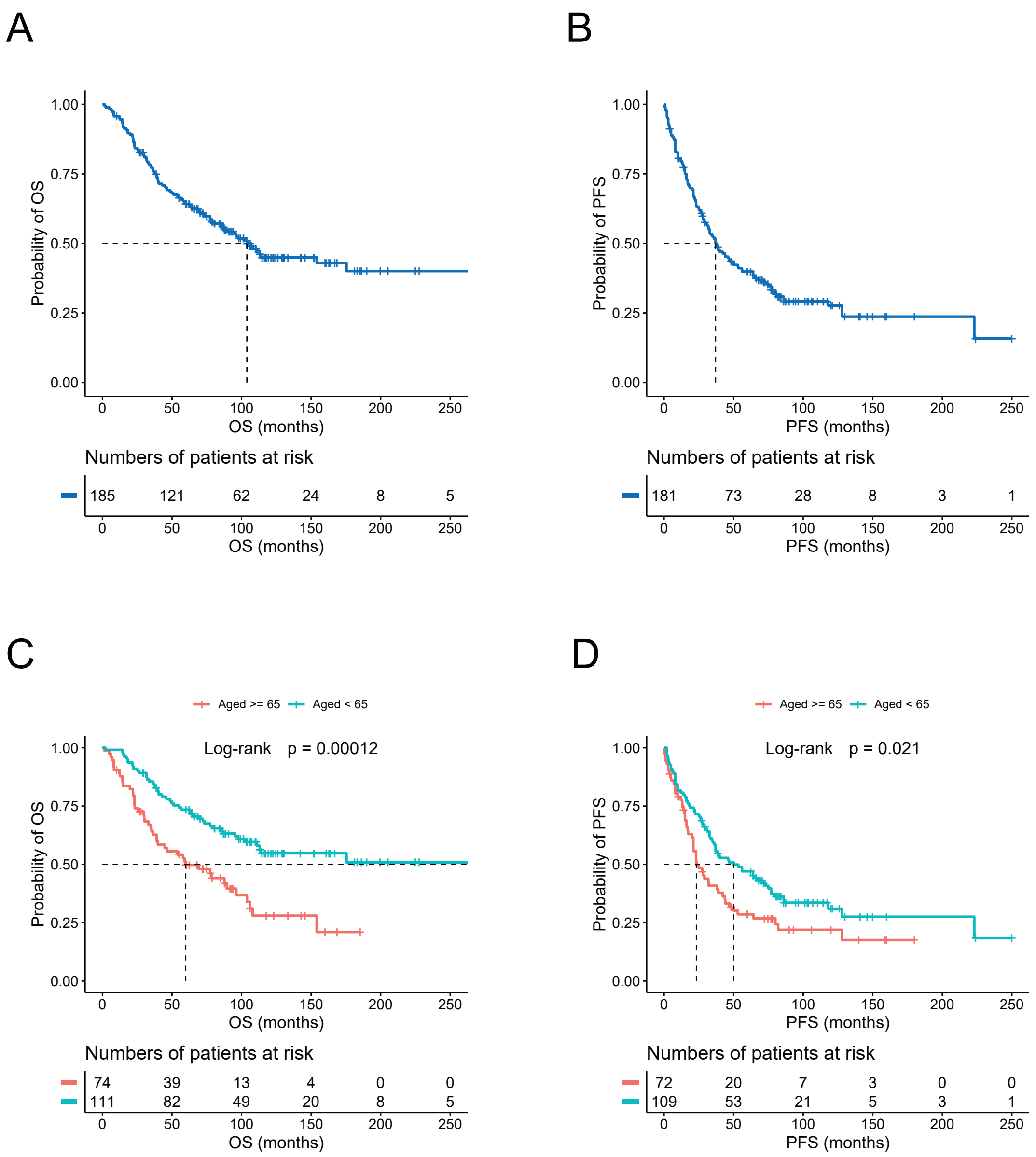

Supplement: Supplementary file 1 — Additional file 1. Supplementary Table S1 and Supplementary Figures. [file 13045_2023_1520_MOESM1_ESM.pdf]
